# *Pygo1 *and *Pygo2 *roles in Wnt signaling in mammalian kidney development

**DOI:** 10.1186/1741-7007-5-15

**Published:** 2007-04-10

**Authors:** Kristopher R Schwab, Larry T Patterson, Heather A Hartman, Ni Song, Richard A Lang, Xinhua Lin, S Steven Potter

**Affiliations:** 1 Division of Developmental Biology, Children's Hospital Medical Center, 3333 Burnet Avenue, Cincinnati, OH 45229, USA; 2 Division of Nephrology, Children's Hospital Medical Center, 3333 Burnet Avenue, Cincinnati, OH 45229, USA; 3 Division of Ophthalmology, Children's Hospital Medical Center, 3333 Burnet Avenue, Cincinnati, OH 45229, USA; 4 Children's Hospital Medical Center, 3333 Burnet Avenue, Cincinnati, OH 45229, USA

## Abstract

**Background:**

The *pygopus *gene of *Drosophila *encodes an essential component of the Armadillo (β-catenin) transcription factor complex of canonical Wnt signaling. To better understand the functions of *Pygopus*-mediated canonical Wnt signaling in kidney development, targeted mutations were made in the two mammalian orthologs, *Pygo1 *and *Pygo2*.

**Results:**

Each mutation deleted >80% of the coding sequence, including the critical PHD domain, and almost certainly resulted in null function. *Pygo2 *homozygous mutants, with rare exception, died shortly after birth, with a phenotype including lens agenesis, growth retardation, altered kidney development, and in some cases exencephaly and cleft palate. *Pygo1 *homozygous mutants, however, were viable and fertile, with no detectable developmental defects. Double *Pygo1*/*Pygo2 *homozygous mutants showed no apparent synergy in phenotype severity. The BAT-gal transgene reporter of canonical Wnt signaling showed reduced levels of expression in *Pygo1*^-/-^/*Pygo2*^-/- ^mutants, with tissue-specific variation in degree of diminution. The *Pygo1 *and *Pygo2 *genes both showed widespread expression in the developing kidney, with raised levels in the stromal cell compartment. Confocal analysis of the double mutant kidneys showed disturbance of both the ureteric bud and metanephric mesenchyme-derived compartments. Branching morphogenesis of the ureteric bud was altered, with expanded tips and reduced tip density, probably contributing to the smaller size of the mutant kidney. In addition, there was an expansion of the zone of condensed mesenchyme capping the ureteric bud. Nephron formation, however, proceeded normally. Microarray analysis showed changed expression of several genes, including *Cxcl13*, *Slc5a2*, *Klk5*, *Ren2 *and *Timeless*, which represent candidate Wnt targets in kidney development.

**Conclusion:**

The mammalian *Pygopus *genes are required for normal branching morphogenesis of the ureteric bud during kidney development. Nevertheless, the relatively mild phenotype observed in the kidney, as well as other organ systems, indicates a striking evolutionary divergence of *Pygopus *function between mammals and *Drosophila*. In mammals, the *Pygo1*/*Pygo2 *genes are not absolutely required for canonical Wnt signaling in most developing systems, but rather function as quantitative transducers, or modulators, of Wnt signal intensity.

## Background

Wnt signaling is of critical importance in several stages of kidney development. Mutual inductive interactions between the ureteric bud and metanephric mesenchyme drive nephrogenesis [1]. The ureteric bud synthesizes Wnt9b, which is essential for induction of the mesenchyme to form nephrons [2]. Wnt4 is made by the induced metanephric mesenchyme and is also required for nephrogenesis [3]. Furthermore, Wnt11, secreted by the ureteric bud tips, participates in a positive feedback loop promoting glial cell line-derived neurotrophic factor (GDNF) expression by the metanephric mesenchyme [4]. Mutations in Wnt9b or Wnt4 result in a dramatic block in nephron formation, while Wnt11 mutants show a significant reduction in nephron number. It is interesting to note that Wnt4 and Wnt11 have been shown to signal, at least in some cases, through noncanonical pathways [5-7], while there is evidence indicating that Wnt9b activates canonical Wnt signaling in the kidney [2].

Genetic studies in *Drosophila *have identified the *Pygopus *(*Pygo*) gene as a critical component of canonical Wnt signaling [8-11]. Pygo and Lgs interact with β-catenin during the formation of the canonical transcriptional complex and are required for accumulation of β-catenin in the nucleus [12]. Lgs binds the central armadillo repeats of β-catenin, while Pygo interacts with Lgs, mediating activation of Wnt targets [9,13]. The N-terminal domain of Pygo is required for Wnt transcriptional activation, while the PHD motif is required for the association of Pygo with Lgs [9,13]. Additionally, a putative nuclear localization signal (NLS) was identified within the N-terminal domain of Pygo, suggesting a possible role of nuclear importation of β-catenin [8,11]. Analyses of multiple aspects of the *pygopus *mutant phenotype indicate that this gene is dedicated to, and required for, canonical Wnt signaling during *Drosophila *development [9].

The mammalian genome carries two, and only two, orthologs of *Drosophila Pygo*, *Pygo1 *and *Pygo2 *[8,9,14]. In this report, we generated targeted mutations of *Pygo1 *and *Pygo2 *to determine their functions, with a particular interest in the contributions of these genes to canonical Wnt signaling during kidney development. The resulting double-homozygous mutant embryos showed a context-dependent reduction in canonical Wnt signaling as measured by Wnt reporter transgene expression. Development remained, however, surprisingly normal, with survival to birth and few apparent defects in most organ systems. Our phenotypic analysis focused on the kidney, which showed altered branching morphogenesis of the ureteric bud, and expansion of the zone of condensed mesenchyme surrounding the ureteric bud, yet relatively normal nephron formation, as measured by histology, confocal analysis, *in situ *hybridization and microarray analysis. The obvious conclusion is that in mammals, unlike *Drosophila*, Pygopus-mediated canonical Wnt signaling is not absolutely necessary in most developing organ systems.

## Results

### *Pygo1 *and *Pygo2 *targeted mutations

We targeted both the *Pygo1 *and *Pygo2 *genes by inserting LoxP sequences to flank critical coding regions including the PHD domains. The resulting targeted mice were mated with transgenic CMV-Cre mice [15] to drive germline LoxP recombination, resulting in the null mutant alleles that were used for this study. PCR confirmed the deletion of the bulk of the coding sequences for both genes, including 89% of coding sequence for *Pygo1 *and 87% of coding sequence for *Pygo2*. Previous studies in *Drosophila *have shown that even a single missense mutation in the PHD domain can eliminate *Pygo *function in Wnt signaling [8].

### *Pygo1 *and *Pygo2 *mutant phenotypes

*Pygo1 *homozygous null mice were viable and fertile with no developmental defects detectable. This was surprising given the importance of the *pygopus *gene in Wnt signaling in *Drosophila*, and the reported expression during development of the mouse *Pygo1 *gene in, for example, the brain, limbs, kidney and branchial arches [14,16] (Yu J, Valerius MT, McMahon AP, contribution to GUDMAP, ).

The *Pygo2 *homozygous null mice survived to birth, but with rare exceptions, died shortly afterwards. The gut, heart and limbs developed without detectable abnormality (data not shown) despite known requirements for Wnt signaling. The *Pygo2 *mutants did, however, show growth retardation, lens agenesis and a kidney phenotype with high penetrance, exencephaly, and cleft palate with incomplete penetrance.

**Figure 1:**
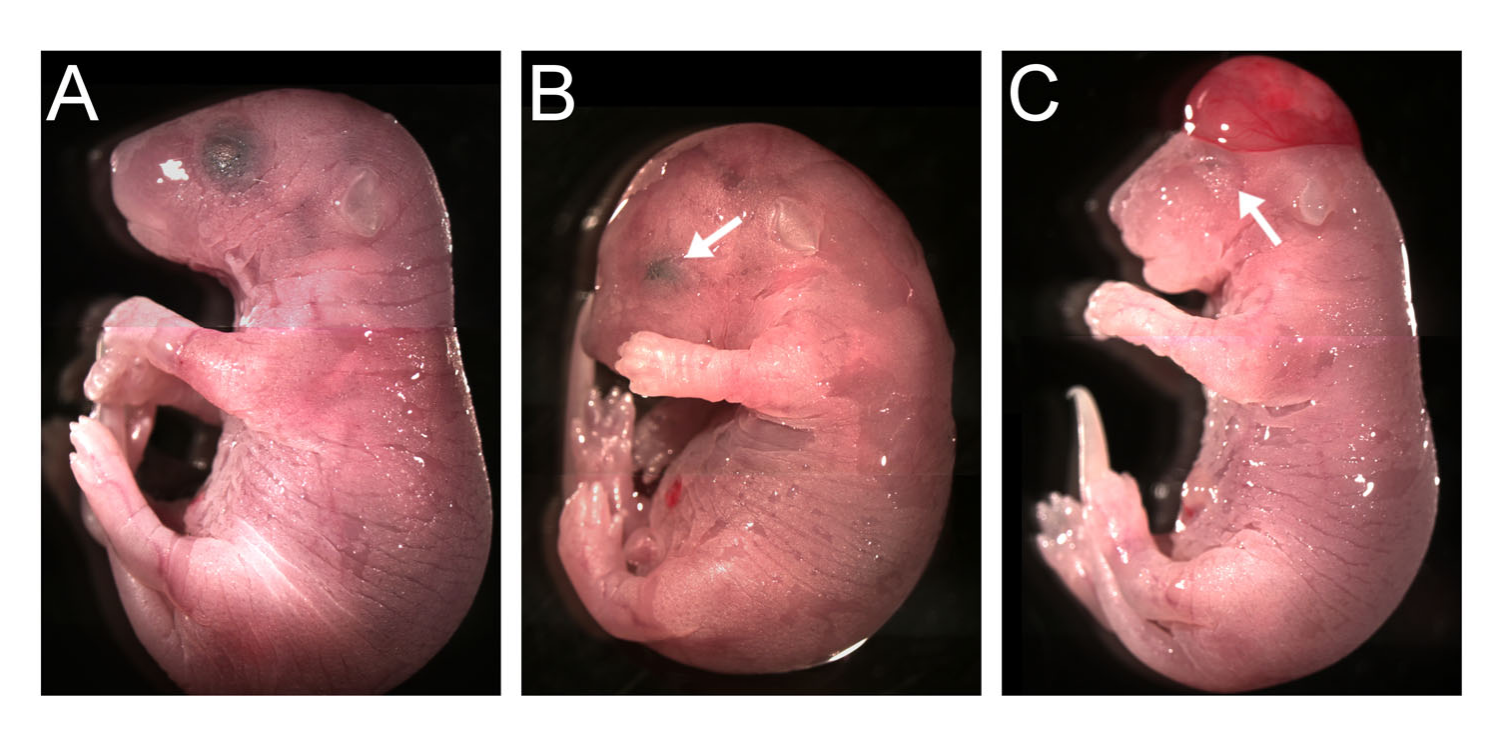
***Pygo1*/*Pygo2 *mutant embryos**. (**A**) E18.5 *Pygo1*^-/-^/*Pygo2*^+/- ^embryos, with only one wild-type *Pygo2 *allele, appeared normal. (**B, C**) Double homozygous mutant *Pygo1*^-/-^/*Pygo2*^-/- ^embryos were smaller, with eye defects including absent or rudimentary lens and folded pigmented retina (arrows). (**C**) A small percentage of *Pygo1*/*Pygo2 *null embryos also displayed exencephaly.

Double-homozygous mutant *Pygo1 *and *Pygo2 *mice had a phenotype similar to that of single *Pygo2 *nulls (Figure 1, Table 1). There was no significant synergism of developmental abnormalities in the double mutants. Together these results suggest that *Pygo2 *is required for the proper development of a limited number of structures, whereas *Pygo1 *is not necessary for normal development.

**Table 1:** Phenotypes of *Pygo2 *wild-type, heterozyogous, and null E18.5 embryos on a *Pygo1 *null background.

	*Pygo2*^+/+^	*Pygo2*^+/-^	*Pygo2*^-/-^
Sample size	6	14	10
Observed phenotypes			
Average weight (g)	1.36	1.33	0.97 *
Cleft palate	0	0	6
Exencephaly	0	0	2
Lens defect	0	0	10
Reabsorption/*in utero *death	0	0	2

* Denotes a significant change (*p *< 0.01) of the average weight of the *Pygo2 *null group compared with both the *Pygo *wild-type and heterozygous groups.

### Expression of *Pygo1 *and *Pygo2 *in the developing kidney

**Figure 2:**
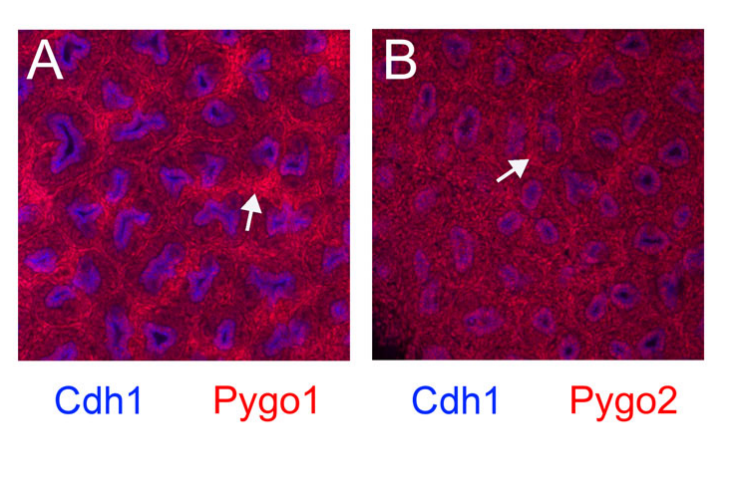
**Pygo1 and Pygo2 expression in the E18.5 developing kidney**. Immunofluorescence was used to determine expression patterns of the Pygo1 and Pygo2 proteins in the cortex of the E18.5 kidney. Both Pygo1 and Pygo2 (red) were localized in the nucleus, as expected. Both genes showed widespread expression, with signal detected in all components of the developing kidney, but with elevated levels in the stromal cell compartment (arrows). Epithelial cells, primarily ureteric bud in these sections, were labeled blue using E-cadherin antibody. Original magnification × 200.

*In situ *hybridization has been used previously to characterize expression of *Pygo1 *[14,16](Yu J, Valerius MT, McMahon AP, contribution to GUDMAP, ). In this report, we used immunofluorescence to better define the expression patterns of the *Pygo1 *and *Pygo2 *genes in the developing kidney. Both genes were widely expressed, showing nuclear localization of encoded proteins in the ureteric bud, capping mesenchyme, and stromal cells (Figure 2). Raised expression of *Pygo1*, and to a lesser degree of *Pygo2*, was seen in stromal cells, and significant nuclear staining was detected in essentially all cells of the developing kidney for both proteins.

### Confocal analysis of *Pygo1/Pygo2 *mutant kidneys

**Figure 3:**
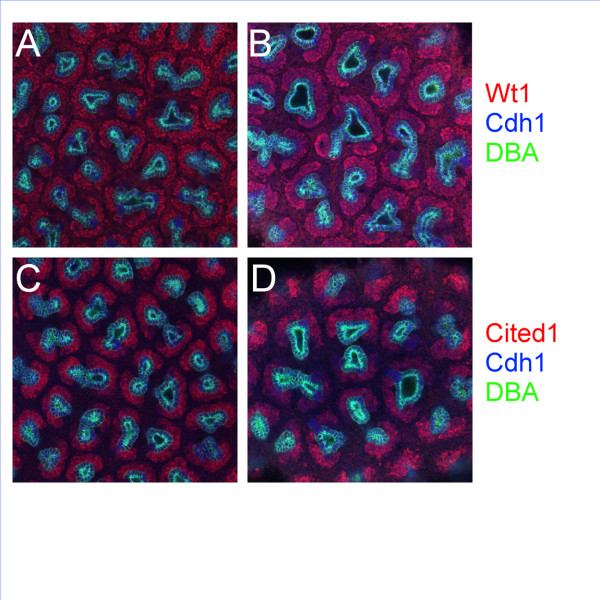
**Confocal analysis of *Pygo1*/*Pygo2 *mutant E18.5 kidneys**. (**B, D**) *Pygo1*^-/-^/*Pygo2*^-/- ^mutant E18.5 kidneys and (**A, C**) kidneys of normal littermates were stained using antibodies to (**C, D**) Cited1 or (**A, B**) Wt1, both expressed in the condensing metanephric mesenchyme, and colored as red, Cdh1, a general marker of epithelia (blue), and DBA lectin staining the ureteric tree [16]. Confocal Z-sections were obtained every 5 μm for 75–80 μm. A reduced number of ureteric tips per area was observed in (**B, D**) the *Pygo1*/*Pygo2 *null kidneys compared with (**A, C**) control littermates. In addition, ureteric bud tips were often more dilated in *Pygo1*/2 null mutants compared with controls. The condensed mesenchyme surrounding the ureteric bud was also significantly expanded in the mutants. Original magnification × 200.

Histological examination of *Pygo2 *null and *Pygo1*/*Pygo2 *double-null mutant kidneys did not reveal any abnormalities in nephrogenesis (data not shown). Confocal analysis was therefore performed to characterize renal development in E18.5 *Pygo1*/*Pygo2 *null kidneys more precisely (Figure 3). Wt1 (red) and Cited1 (red) antibodies both stain the capping metanephric mesenchyme around the ureteric bud tips [17]. WT1 antibody also stains renal vesicles and glomerular anlage. Antibodies to Cdh1, also known as E-cadherin (blue), were used to identify epithelial structures, including the branching ureteric bud and nascent nephrons of the developing kidney [18]. *Dolichos biflorus *(DBA) lectin [16] was used to selectively stain ureteric bud-derived structures [19].

Wt1 and Cited1 (red) staining revealed an increase of approximately 30% in the thickness of the capping mesenchyme surrounding the mutant ureteric buds (Figure 3, Table 2). Nevertheless, the mutant metanephric mesenchyme underwent relatively normal nephrogenesis. Cdh1-staining nephrons (blue) were identified connecting to the ureteric bud tips [16] and extending into the medulla of the kidney, as normal. Intermediate structures of nephrogenesis, including renal vesicles, comma-shaped bodies, and S-shaped bodies appeared normal.

**Table 2:** Confocal analysis of *Pygo2 *wild-type, heterozygous, and null E18.5 kidneys on the *Pygo1 *null background.

Genotype	Ureteric tip			Condensing mesenchyme		
	
	Ureteric tips/mm^2^	SD	*n*	Mesenchyme condensate size (μm)	SD	*n*
*Pygo2*^+/+^	196	8.36	3	16.2	1.09	3
*Pygo2*^+/-^	191	11.6	7	15.6	1.18	5
*Pygo2*^-/-^	147*	12.2	7	20.9 *	1.12	5

*Significant change (P < 0.01) between the three groups.

SD, Standard deviation; n, number

Confocal analysis also showed that the ureteric bud tips [16] of *Pygo1*/*Pygo2 *null kidneys were dilated and misshaped compared with those of littermates with at least one wild-type *Pygo2 *allele (Figure 3). In addition, the *Pygo1*/*Pygo2 *double-homozygous mutant kidneys also had a decrease of approximately 25% in the number of ureteric bud tips per area compared with littermates with at least one wild-type copy of *Pygo2 *(Table 2). No significant difference in ureteric bud-tip density was seen between *Pygo2*^+/- ^and *Pygo2*^+/+ ^embryos (P = 0.33).

### *In situ *hybridization

**Figure 4:**
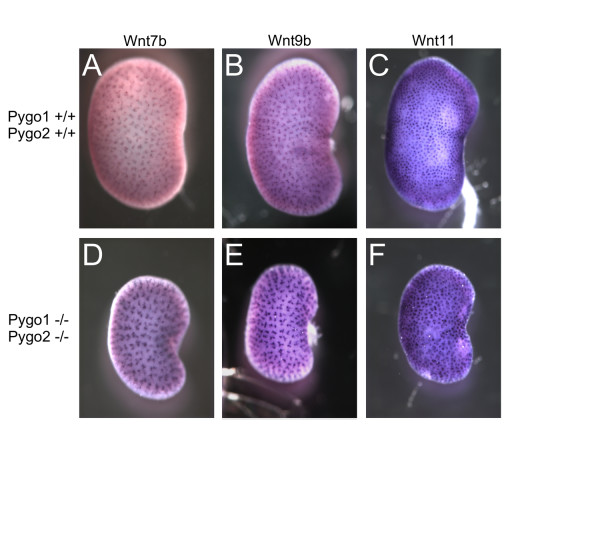
**Expression of *Wnt7b*, *Wnt9b*, and *Wnt11 *in E18.5 *Pygo1*/*Pygo2 *compound null kidneys**. Whole-mount *in situ *of hybridizations of (**A-C**) E18.5 wild-type kidneys and (**D-F**) *Pygo1*^-/-^/*Pygo2*^-/- ^mutant kidneys with the ureteric bud derivative markers: (**A, D**) *Wnt7b*, (**B, E**) *Wnt9b*, and (**C, F**) *Wnt11*. The mutant kidneys showed normal expression patterns for the ureteric stalk markers *Wnt7b *and *Wnt9b*, and reduced density of ureteric tips as measured by *Wnt11*. Original magnification × 32.

We examined expression of three *Wnt *genes in *Pygo *mutants. *Wnt7b *is expressed in the stalks [20], *Wnt11 *in the tips [4], and *Wnt9b *in the stalks and weakly in the tips of the branching ureteric bud [2]. All three genes showed similar expression patterns in *Pygo2*^+/+ ^and *Pygo1*/*Pygo2 *double-mutant kidneys (Figure 4). In addition, these *in situ *hybridization patterns confirmed the confocal microscopy results, showing a reduced number of tips (Wnt11-positive) per surface area in the *Pygo1*/*Pygo2 *double-homozygous mutants.

### Reduced canonical Wnt signaling in *Pygo1/Pygo2 *mutant mice

**Figure 5:**
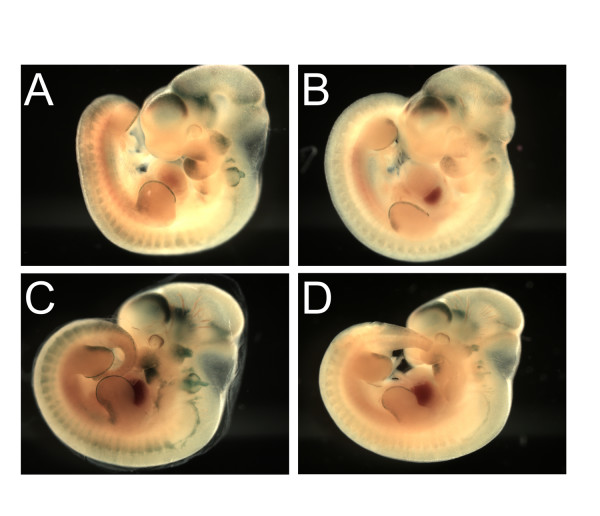
**Reduced canonical Wnt signaling in *Pygo2 *and *Pygo1*/*Pygo2 *mutant embryos**. E10.5 embryos, all with the BAT-gal transgene reporter of canonical Wnt signaling. (**A**) *Pygo1*^+/+^/*Pygo2*^+/- ^embryos showed normal X-gal staining in the developing brain, pharyngeal pouches, otic vesicle, apical ectodermal ridges of the fore and hind limb buds, and in somites. (**B**) *Pygo1*^+/-^/*Pygo2*^-/- ^embryos, with loss of both *Pygo2 *alleles, showed reduced but not absent BAT-gal reporter expression in many developing structures, including pharyngeal pouches, otic vesicle, and somites. (**C**) *Pygo1*^-/-^/*Pygo2*^+/- ^embryo, with mutation of both *Pygo1 *alleles, but one wild-type *Pygo2 *allele, showed normal BAT-gal expression. (**D**) Double-homozygous mutant *Pygo1*^-/-^/*Pygo2*^-/- ^embryos still showed some remaining BAT-gal expression, suggesting residual canonical Wnt signaling. Embryos in panels (**A**) and (**B**) were from the same litter and were processed in parallel, while embryos in (**C**) and (**D**) were from a separate litter, also processed in parallel, and were slightly more developmentally advanced. Original magnification (A, B) × 20; (C, D) × 16.

The BAT-gal transgene reporter of canonical Wnt signaling [21] was used to examine changes in Wnt signaling in the mutant mice. Both *Pygo2*^-/- ^and *Pygo1*/*Pygo2 *double-null E10.5 embryos showed a decrease in canonical Wnt signaling (Figure 5). There was, however, tissue-specific variability in the degree of reduction, with the somites, for example, showing strong reduction, while the mutant telencephalon still showed robust Wnt signaling. These results suggest that the mammalian *Pygo *genes are significant modulators of canonical Wnt signaling in some, but not in all developing systems.

**Figure 6:**
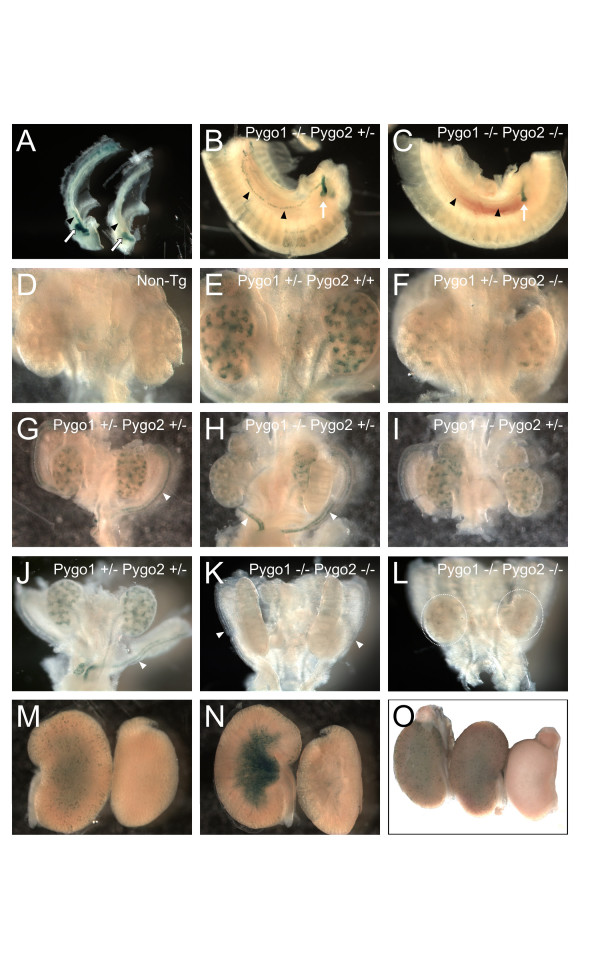
***Pygo2 *is required for BAT-gal reporter expression in ureteric bud-derived structures of the developing kidney**. X-Gal staining of BAT-gal transgenic (**A-C**) E10.5, and (**E-L**) E13.5 urogenital tracts, and (**M-O**) E18.5 kidneys. (**A**) *Pygo1*^-/-^/*Pygo2*^+/+ ^(left) and *Pygo1*^+/-^/*Pygo2*^-/- ^(right). Note the loss of reporter activity in the nephric duct (black arrowhead) and reduction of staining in the ureteric bud (white arrow) of the *Pygo2 *null embryo (Right). (**B**) *Pygo1*^-/-^/*Pygo2*^+/- ^E10.5 embryo with BAT-gal reporter activity in the nephric duct (black arrowhead) and ureteric bud (white arrow). (**C**) *Pygo1*^-/-^/*Pygo*^-/- ^embryo, with reporter expression lost in the nephric duct and reduced in the ureteric bud (white arrow). (**D**) Control background X-gal staining of a urogenital tract from an E13.5 embryo without the BAT-gal transgene (Non-Tg), showing absence of endogenous beta-galactosidase activity. (**E**) *Pygo1*^+/-^/*Pygo2*^+/+^, with BAT-gal reporter activity in the ureteric compartment of the developing kidney, including the ureteric tips, ureteric tree, and ureter. (**F**) *Pygo1*^+/-^/*Pygo2*^-/-^, with marked reduction of BAT-gal reporter activity in the ureteric compartment. (**G**) *Pygo1*^+/-^/*Pygo2*^+/-^, with reporter expression in the paramesonephric duct (white arrowhead) and ureteric tree. (**H, I**) *Pygo1*^-/-^/*Pygo2*^+/-^, with (**H**) ventral view showing reporter expression in the paramesonephric duct (white arrowhead), and (**I**) dorsal view showing ureteric tree expression in the kidney. (**J**)*Pygo1*^+/-^/*Pygo2*^+/-^, a control processed in parallel with (**K**) and (**L**), with expression in ureteric tree and paramesonephric duct. (**K, L**)*Pygo1*^-/-^/*Pygo2*^-/-^, reporter activity was lost in the paramesonephric duct (white arrowhead), and in the ureter and ureteric compartment of the developing kidneys (dashed circles), except for (**K**) a few faintly staining cells. (**M, N**) *Pygo1*^+/-^/*Pygo2*^+/- ^(left) and *Pygo1*^-/-^/*Pygo2*^-/- ^(right) E18.5 kidneys. The kidneys in (**N**) were bisected. BAT-gal reporter expression was seen in the ureteric tree components of the cortex and medulla of the double heterozygotes but was almost completely lost in the double-homozygous mutants. (**O**) *Pygo1*^+/-^/*Pygo2*^+/+ ^(left)*, Pygo1*^-/-^/*Pygo2*^+/- ^(middle), and *Pygo1*^-/-^/*Pygo2*^-/- ^(right) E18.5 kidneys. Reporter activity was present in the ureteric compartments of the *Pygo1*^+/-^/*Pygo2*^+/+ ^(left) and *Pygo1*^-/-^/*Pygo2*^+/- ^(middle) kidneys, but lost in the *Pygo1*^-/-^/*Pygo2*^-/- ^(right) kidney. Original magnification: (**A-C**) × 32, (**D-F**) × 63, (**G-I**) × 40, (**J-L**) × 50, (**M, N**) × 10, and (**O**) × 12.5.

We also examined BAT-gal reporter expression in more detail in the developing urogenital system of *Pygo *mutants. The results suggested that the *Pygo2 *gene is required for canonical Wnt signaling in the nephric duct. Both *Pygo2 *null and *Pygo1*/*Pygo2 *double-null E10.5 embryos showed an absence of reporter expression in the nephric duct, while control littermates showed strong expression (Figure 6A–C). In *Pygo1*/*Pygo2 *double-null mutants, the nephric duct did form, however, and give rise to the ureteric bud outgrowth, which showed reduced but not absent BAT-gal reporter expression (Figure 6A–C).

At E13.5, the *Pygo2 *gene appeared to play a major role, and the *Pygo1 *gene a minor role, in canonical Wnt signaling in the ureteric tree, as measured by BAT-gal expression. A negative control kidney, without the BAT-gal transgene, showed minimal background X-gal staining (Figure 6D), whereas a BAT-gal transgenic kidney with at least one wild-type *Pygo2 *gene showed strong X-gal staining in the ureteric tree (Figure 6E). In contrast, *Pygo1*^+/-^/*Pygo2*^-/- ^E13.5 kidneys showed very weak reporter expression (Figure 6F), suggesting a significant loss of canonical Wnt signaling. Homozygous loss of the *Pygo1 *gene alone, however, had a small effect on reporter expression (Figure 6G–H). Homozygous mutation of both the *Pygo1 *and *Pygo2 *genes gave a more dramatic reduction of BAT-gal expression than loss of *Pygo2 *alone (Figure 6F, L).

The *Pygo1 *and *Pygo2 *genes were also required for BAT-gal reporter expression in the paramesonephric (Mullerian) ducts. In *Pygo2*^-/- ^mice, there was a significant loss of reporter expression (data not shown), and double-homozygous mutants showed loss of X-gal staining in the paramesonephric ducts (Figure 6K), whereas *Pygo1*^-/-^/*Pygo2*^+/- ^and *Pygo1*^+/-^/*Pygo2*^+/- ^mice showed normal levels of BAT-gal expression (Figure 6G, H, J).

BAT-gal reporter analysis of the *Pygo *mutants at a later developmental stage, E18.5, also identified a significant decrease in canonical Wnt signaling in the cortical ureteric branches and renal pelvis of the developing kidney (Figure 6M–O). Cortical X-gal staining was seen in the ureteric branches of a *Pygo1*^+/-^/*Pygo2*^+/- ^kidney (Figure 6M, left), but was completely absent in the cortex of a *Pygo1*^+/-^/*Pygo2*^-/- ^kidney (Figure 6M, right). Bisection revealed a significant loss of X-gal staining cells in the collecting ducts and renal pelvis of the *Pygo2 *null kidney compared with control littermates (Figure 6N). Side by side comparison of *Pygo1*^+/+^/*Pygo2*^+/- ^(Figure 6O, left), *Pygo1*^-/-^/*Pygo2*^+/- ^(Figure 6O, middle), and *Pygo1*^-/-^/*Pygo2*^-/- ^(Figure 6O, right) E18.5 kidneys suggested a significant role for the *Pygo2 *gene in canonical Wnt signaling in the ureteric tree and its derivatives.

**Figure 7:**
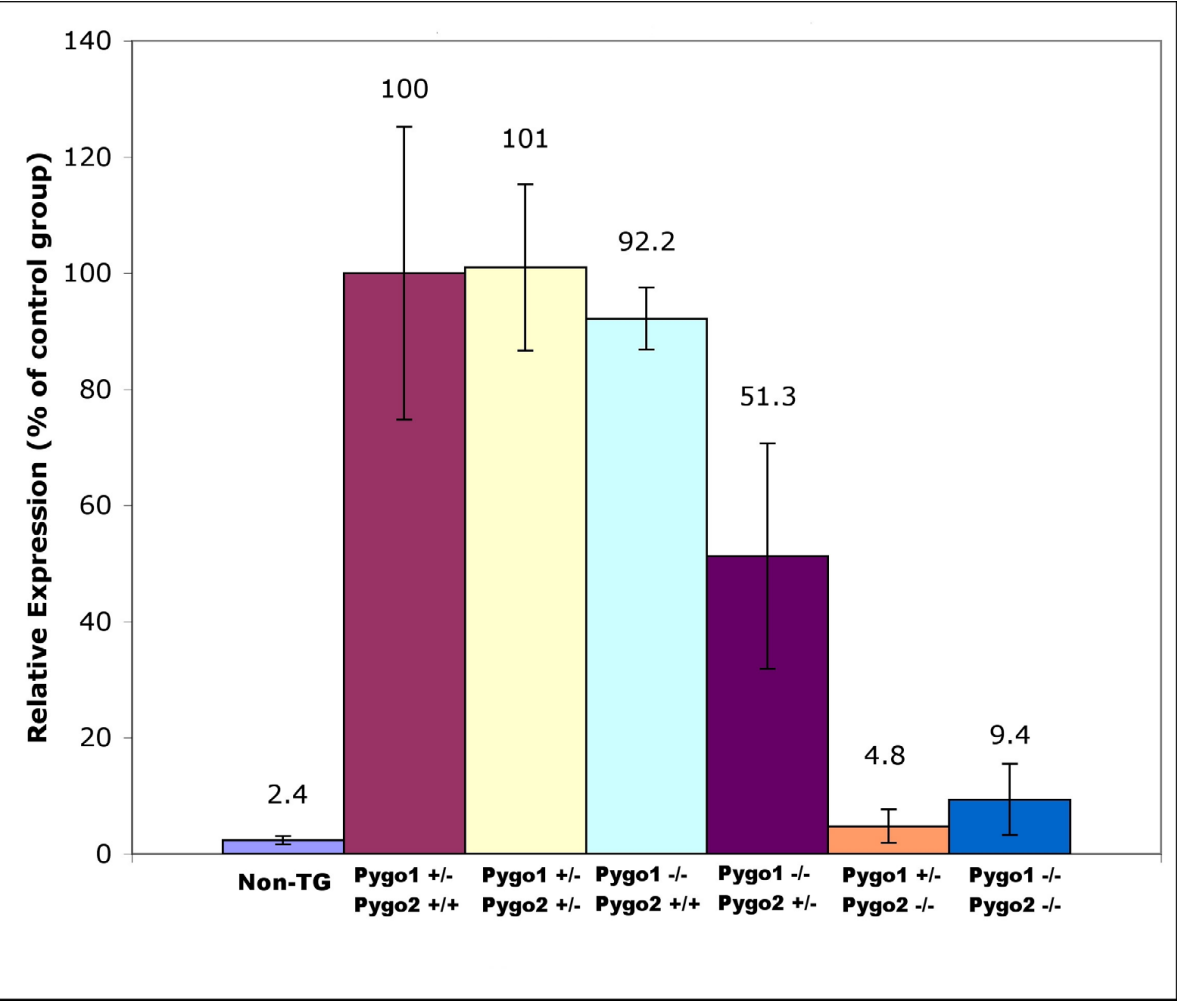
**Quantitative analysis of BAT-gal reporter expression in *Pygo1*/*Pygo2 *E18.5 kidney extracts**. Transgene-specific beta-galactosidase was quantified by ELISA analysis. *Pygo1*^+/-^/*Pygo2*^+/+^, *Pygo1*^+/-^/*Pygo2*^+/- ^and *Pygo1*^+/-^/*Pygo2*^+/+ ^kidneys all showed similar levels of BAT-gal expression. In the *Pygo2 *hetereozygote, *Pygo1 *homozygous mutants, however, reporter expression was reduced by about 50%. In *Pygo2 *homozygous mutants BAT-gal expression was uniformly low, with or without wild-type *Pygo1 *alleles. Each experimental group included a sample size of at least four.

In order to validate and quantify the BAT-gal reporter expression changes in the *Pygo2 *null and *Pygo1*/*Pygo2 *nulls, we performed ELISA measurements of transgene specific β-galactosidase levels in E18.5 kidneys (Figure 7). Loss of the *Pygo2 *gene (*Pygo1*^+/-^/*Pygo2*^-/- ^and *Pygo1*^-/-^/*Pygo2*^-/-^) gave greater than 90% reduction in BAT-gal expression. Loss of *Pygo1 *alone (*Pygo1*^-/-^/*Pygo2*^+/+^) did not result in a significant change. Interestingly, however, the *Pygo1*^-/-^/*Pygo2*^+/- ^showed only 50% of wild-type BAT-gal expression, suggesting a minor contribution by *Pygo1 *in canonical Wnt signaling. Although BAT-gal expression was decreased in the E18.5 *Pygo1*^-/-^/*Pygo2*^+/- ^kidney, confocal analysis of kidneys with this genotype revealed no dilated ureteric tips or significant changes in ureteric tip number per area compared with *Pygo1*^-/-^/*Pygo2*^+/+ ^kidneys (data not shown). Collectively, these BAT-gal reporter results suggest a significant role for the *Pygo1 *and *Pygo2 *genes in canonical Wnt signaling during development of the ureteric tree of the kidney.

Interestingly, however, even in wild-type mice the BAT-gal reporter showed no expression in the developing metanephric mesenchyme, or metanephric mesenchyme-derived structures, such as renal vesicles, S-shaped bodies, tubules, and glomeruli. This has been reported previously, and was interpreted to indicate the absence of canonical Wnt signaling in the metanephric mesenchyme [21]. Results from other studies, however, argue for the presence of canonical Wnt signaling in the metanephric mesenchyme. For example, ureteric bud expression of Wnt1, thought to act through canonical Wnt signaling, can rescue *Wnt9b *mutants and induce nephrogenesis of the metanephric mesenchyme [2]. This suggests that the BAT-gal transgene might not accurately report canonical Wnt signaling in the metanephric mesenchyme. To address this question, we incubated E11.5 metanephric mesenchyme in lithium chloride (LiCl), which activates canonical Wnt signaling through inhibition of GSK3, and also functions as an inducer of nephrogenesis in kidney organ culture [22]. We observed that LiCl-treated metanephric mesenchyme did undergo nephrogenesis, as expected, but failed to show BAT-gal expression (data not shown), suggesting that this transgene is not an accurate reporter of canonical Wnt signaling in the metanephric mesenchyme of the developing kidney.

### Microarray analysis of *Pygo1/Pygo2 *null kidneys

To further examine possible disturbance of gene expression in the *Pygo1*/*Pygo2 *mutant kidneys, we used microarrays to perform a global analysis of gene expression changes. Whereas the BAT-gal transgene reporter monitors the response of one promoter to Wnt signaling, microarrays can be used to follow expression changes of all genes, including all known Wnt targets. E18.5 wild-type and *Pygo1*^-/-^/*Pygo2*^-/- ^kidneys were examined in biological triplicate. In total, 45 genes were identified as significantly changed, using a relatively low stringency screen of the data, with a *p*-value cutoff of <0.05, and fold change >2 (Table 3). Notably, both *Pygo1 *and *Pygo2 *were identified as downregulated (- 5.3-fold and - 3.9-fold, respectively) in the mutant kidneys. Other genes with significant decreases in mutant kidneys included *Cxcl13 *(- 2.3-fold), *Slc5a2 *(- 2.8-fold), and *Slco1a4 *(- 2.1-fold). *Slc5a2 *is expressed in the proximal tubules of the adult nephron and has been implicated in autosomal recessive renal glucosuria, characterized by loss of glucose uptake by the nephron [23]. The organic anion transporter, *Slco1a4*, is also strongly expressed in the tubules of the adult kidney [24]. These results suggest that the *Pygo1*/*Pygo2 *genes might play a role in nephron maturation and subsequent function.

**Table 3:** Genes differentially expressed (*p *< 0.05, two-fold change or greater) in the *Pygo1*/*Pygo2 *null E18.5 kidney normalized to wild-type samples.

Gene symbol	Gene name	*Pygo1*/*2 *null average fold change
*C920006O11Rik*	Hypothetical protein	7.1
*Tia1*	Cytotoxic granule-assoc RNA binding 1	6.6
*Akr1e1*	Aldo-keto reductase family 1, E1	5.5
*5830417I10Rik*	Hypothetical protein	3.8
*XM_193262*	Protein 40kD (LOC269251) mRNA.	3.8
*Picalm*	Pl-binding clathrin assembly protein	3.1
*Pck1*	PEP carboxykinase 1, cytosolic	2.9
*Klk5*	Kallikrein 5	2.8
*Klk6*	Kallikrein 6	2.7
*AK051496*	4933409K07Rik protein	2.5
*Timeless*	Timeless homolog	2.4
*Klk27*	Kallikrein 27	2.4
*Gsta2*	Glutathione S-transferase, alpha 1	2.4
*Tia1*	cytotoxic granule-assoc RNA binding 1	2.3
*AK049070*	Hypothetical protein	2.3
*BC051083*	Cisplatin resistance associated	2.3
*1500015O10Rik*	Esophageal cancer related gene 4	2.3
*Klk27*	Kallikrein 27	2.2
*Npy*	Neuropeptide Y	2.2
*Ren2*	Renin 2	2.2
*Gsta2*	Glutathione S-transferase, alpha 2	2.1
*Col8a1*	Procollagen, type VIII, alpha 1	2.1
*Col8a1*	Procollagen, type VIII, alpha 1	2.1
*G6pc*	Glucose-6-phosphatase, catalytic	2.1
*Rgs4*	Regulator of G-protein signaling 4	2.0
*XM_358675*	Hypothetical protein	2.0
*Ctse*	Cathepsin E preproprotein	- 2.0
*Masp2*	Mannan-binding lectin serine protease 2	- 2.0
*Slco1a4*	Solute carrier organic anion transporter 1a4	- 2.1
*Cyp2e1*	Cytochrome P450 2E1	- 2.1
*Sah*	SA hypertension-associated homolog	- 2.1
*Aldh1a7*	Aldehyde dehydrogenase 1A7	- 2.2
*1200006F02Rik*	Hypothetical protein	- 2.3
*2600001B17Rik*	Hypothetical protein	- 2.3
*D630004N19Rik*	Hypothetical protein	- 2.3
*Cxcl13*	Chemokine (C-X-C motif) ligand 13	- 2.3
*Hpgd*	Hydroxyprostaglandin dehydrogenase 15	- 2.4
*Lum*	Lumican	- 2.5
*Pvalb*	Parvalbumin	- 2.5
*Cml4*	N-acetyltransferase Camello 4	- 2.7
*Slc5a2*	Solute carrier 5 (sodium/glucose) 2	- 2.8
*Pygo2*	Pygopus 2	- 3.9
*Pygo1*	Pygopus 1	- 5.3
*2610305D13Rik*	Hypothetical protein	- 5.6
*Csrp1*	Cysteine and glycine-rich protein 1	- 7.1

Noteworthy genes upregulated in the *Pygo1*/*Pygo2 *null kidney included *Klk5 *(2.8-fold), *Klk6 *(2.7-fold), *Ren2 *(2.2-fold), and *Timeless *(2.4-fold). *Klk5 *and *Klk6 *are members of the kallikrein family of trypsin-like serine proteases. Kallikreins have diverse functions in cancer, tissue remodeling, and regulation of blood pressure [25]. *Ren2*, a homolog of the endopeptidase *Renin 1*, activates the renin-angiotensin system, increasing blood pressure [26]. Disruption of the renin-angiotensin system during development results in congenital abnormalities of the ureter and collecting-duct system [27]. *Timeless*, a transcription factor involved in the regulation of circadian rhythms, and expressed in the branching ureteric tips, has also been shown to regulate ureteric branching morphogenesis [28].

**Figure 8:**
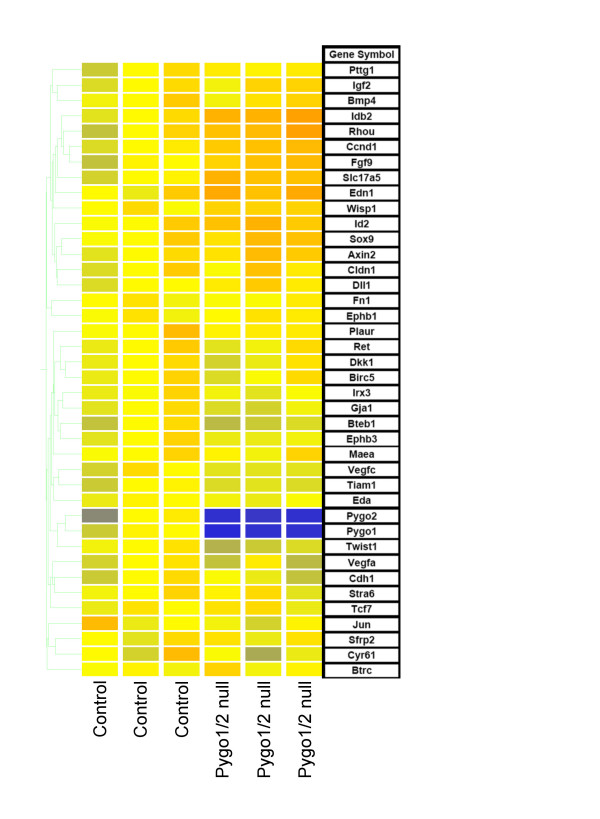
**Gene expression changes of common Wnt signaling targets in the E18.5 *Pygo1*/*Pygo2 *null kidney**. Microarray analysis was performed in triplicate on wild-type and *Pygo1*/2 compound null E18.5 kidneys. Possible Wnt targets were selected from those compiled at [39]. An initial gene list of 82 Wnt targets was created and then reduced to a total of 40 genes using an expression level restriction requiring the raw expression intensity to be >100 in at least 3 samples. *Pygo1 *and *Pygo2 *probes were included to demonstrate significant changes in expression levels.

We were interested in the possible altered expression of previously known Wnt targets in the *Pygo1*^-/-^/*Pygo2*^-/- ^mutant kidneys. A list of known Wnt targets was compiled from . Both *Pygo1 *and *Pygo2 *probe sets were included as references to illustrate significant down regulation. Remarkably, the known Wnt target genes showed very few differences in expression between wild-type and mutant kidneys (Figure 8). Only the expression of *Ccnd1 *encoding cyclin D1 [29], and *Wisp1 *[30] were significantly changed, with expression level changes of <1.5-fold in *Pygo1*/*Pygo2 *null kidneys. These results indicate an absence of dramatic changes in expression of previously known Wnt signaling target genes in the *Pygo1*/*Pygo2 *mutant kidneys.

We used quantitative real-time PCR, with independent biological samples, to validate the microarray results. Nine genes were tested, and for seven we did observe a significant fold change in the same direction predicted by the microarray results (Table 4). It is not surprising that two of the nine genes could not be validated, considering the relatively low stringency used in screening the microarray data.

**Table 4:** Validation of gene expression changes in the E18.5 *Pygo1*/*Pygo2 *null kidneys normalized to E18.5 wild-type kidneys.

Gene symbol	Microarray fold change	QPCR fold change (SD)	Validation
Aldh1a7	- 2.2	1.1 (0.11)	No
Col8a1	2.1	2.9 (0.78)	Yes
Csrp1	- 7.1	1.2 (0.02)	No
Klk5	2.8	5.8 (0.74)	Yes
Pygo2	- 3.9	- 3.7 (0.04)	Yes
Picalm	3.1	2.2 (0.24)	Yes
Ren2	2.2	6.1 (0.36)	Yes
Tia1	6.6 and 2.3	Amplification not detected in wild-type samples	Yes

## Discussion

In *Drosophila*, the *Pygopus *gene is a key mediator of canonical Wnt signaling. In one study, 12 distinct measures of Wnt signaling in *Pygo *mutants were performed, including analysis of leg, wing, and eye imaginal discs. In 2 cases there was a significant reduction of Wnt signaling and in 10 cases a complete block [10]. A second study in *Drosophila *examined the effects of *Pygo *mutation on cuticle patterning, midgut constriction, central nervous system, and cardiac development, and concluded that "*Pygo *is an essential component in the Wg signal transduction pathway". [8].

Given these results in *Drosophila*, the relatively mild phenotypes of mice with targeted *Pygo1*, *Pygo2*, or double *Pygo1*/*Pygo2 *mutations were striking. *Pygo2 *mutants developed to birth and showed limited abnormalities, while *Pygo1 *homozygous mutants were normal and fertile. Furthermore, there was no detected synergism in the phenotype of the double *Pygo1*/*Pygo2 *mutant, although in several tissues the BAT-gal Wnt reporter did show more a severe reduction in expression. One possible explanation of these unexpected results is a failure of the gene targeting to eliminate functioning of the *Pygo1 *and *Pygo2 *genes. The *Pygopus *deletion alleles described in this report, however, are almost certainly functional nulls. In *Drosophila*, it has been shown that the PHD domain is absolutely necessary for Pygopus function in Wg signaling. The PHD domain is 60 amino acids with seven cysteines and a histidine, predicted to chelate two zinc ions. PHD domains are found in diverse proteins, including transcription factors, and have been implicated in chromatin remodeling and protein-protein interactions [8]. In *Drosophila *the *pygo*^F107 ^allele, with a single missense mutation converting amino acid 802 in the PHD domain from cysteine to tyrosine, loses Wnt signaling function in both embryogenesis and imaginal disc development [8]. The *Pygo1*/*Pygo2 *mutant alleles made in this report carried deletions of the entire PHD domains, as well as most other coding sequences. For the *Pygo1 *gene, the coding region for 372 of 417 total amino acids was deleted, and for the *Pygo2 *gene, we deleted coding for 354 of 405 amino acids. It is therefore very unlikely that the relatively mild phenotypes observed were the result of residual function of the targeted alleles.

We focused our analysis on the developing kidney, in which Wnt signaling has been shown to be of critical importance in several stages of nephrogenesis. Wnt9b is made by the ureteric bud and induces the metanephric mesenchyme to undergo nephrogenesis [2]. Downstream of Wnt9b is Wnt4 [2], which is made by the metanephric mesenchyme, and is also required for nephrogenesis [3]. In addition, Wnt11 is produced by the ureteric bud tips and induces GDNF expression in the metanephric mesenchyme [4].

In this report we describe a novel Wnt function in kidney development. The BAT-gal transgene reporter indicated the presence of canonical Wnt signaling in the ureteric bud and its derivatives in the developing kidney. Further, in the *Pygo2 *mutants this signal was lost, suggesting significant reduction of Wnt signaling. In addition, we observed a resulting decrease in ureteric tip density, reduced kidney size and altered morphology of the ureteric tree in mutants, indicating a role for canonical Wnt signaling in branching morphogenesis of the ureteric bud. While the simplest interpretation is direct Wnt signaling to the ureteric bud, it remains possible that the observed abnormalities are the result of indirect effects, with altered Wnt signaling to the metanephric mesenchyme then affecting mesenchyme to ureteric bud signaling.

The *Pygo1*/*Pygo2 *mutant phenotype of reduced branching morphogenesis of the ureteric bud is surprisingly similar to that previously reported for *Wnt11 *mutants [4]. The underlying mechanisms, however, are likely to be distinct. Wnt11 is generally thought to act through a noncanonical pathway, (although exceptions have been noted [31]), whereas the *Pygopus *genes promote canonical Wnt signaling. Further, the *Wnt11 *mutants showed an altered feedback loop between Wnt signaling from the ureteric bud to the mesenchyme and GDNF signaling from the mesenchyme to the bud, whereas in the *Pygo1*/*Pygo2 *mutants, we observed disrupted Wnt signaling in the ureteric bud.

The *Pygo1*/*2 *mutants also showed an expansion of the zone of thickened mesenchyme that caps the ureteric bud. Nevertheless, the mutant metanephric mesenchyme formed nephrons normally. This was particularly interesting, as Wnt9b signaling from the ureteric bud to the metanephric mesenchyme has been shown to induce nephrogenesis via canonical Wnt signaling [2].

The results in this report indicate a striking and surprising evolutionary divergence of *Pygopus *function between *Drosophila *and mammals. In *Drosophila*, the *Pygopus *gene is often required for canonical Wnt signaling, while in mammals the *Pygo1*/*Pygo2 *genes appear to play a smaller role in canonical Wnt signaling. The BAT-gal transgene reporter of canonical Wnt signaling showed reduced but generally not absent signal in *Pygo1*/*Pygo2 *mutant embryos, with tissue-specific variation in level of diminution. In addition, the kidneys were not unique in showing surprisingly normal development in *Pygo1*/*Pygo2 *mutants. Indeed, organogenesis generally proceeded without detectable abnormality, with few exceptions. These results suggest that the proteins encoded by mammalian *Pygopus *genes are often mere modulators of canonical Wnt signaling intensity, and not essential components.

The microarray results further support this conclusion. Whereas the BAT-gal transgene reporter monitors the expression level of only one Wnt responsive promoter, the microarray allows the analysis of the activity levels of promoters of all genes. Interestingly, the mutant kidneys showed gene-expression profiles surprisingly similar to wild type. Some genes did, however, show expression differences, and a high percentage of these differences could be validated by real-time PCR with independent biological samples. Assuming that the *Pygopus *genes in mammals are dedicated to canonical Wnt signaling, as has been previously shown in *Drosophila*, the genes with expression differences represent candidate Wnt targets (direct or indirect) in kidney development. We would predict these genes to show greater changes in expression level in a developing kidney with a more complete removal of canonical Wnt signaling.

## Conclusion

In conclusion, the mammalian *Pygo2 *gene is required for normal branching morphogenesis of the ureteric bud, with mutants showing dilated tips and reduced numbers of tips. In addition, in *Pygo2 *mutants there was an expansion of the zone of metanephric mesenchyme that caps the ureteric buds. Nevertheless, nephron formation proceeded remarkably normally, even in *Pygo1*/*Pygo2 *double-homozygous mutants. This was surprising considering the importance of the *Pygopus *gene in canonical Wnt signaling in *Drosophila*, and the importance of canonical Wnt signaling in nephrogenesis. The results argue that the mammalian *Pygopus *genes are, in most developing systems, only quantitative transducers of Wnt signaling. Previous cell culture studies [32,33] and the reduced BAT-gal transgene reporter expression in the *Pygo1*/*Pygo2 *knockout mice described in this report, do confirm an evolutionarily conserved function in canonical Wnt signaling. In mammals, however, the phenotypic effects of *Pygopus *mutation are much milder than in *Drosophila*. The degree of importance of the *Pyg1*/*2 *genes in Wnt signaling was context-dependent, but in general, mammalian organogenesis remained intact in *Pygopus *mutants. Perhaps the simplest explanation is that in mammals, other genes show partial functional redundancy with the two *Pygopus *genes. The β-catenin transcription-factor complex includes a large and growing number of proteins , some of which may share the nuclear localization and/or transcription activation functions of *Pygo1*/*Pygo2 *in mammals. The identities and roles of these *Pygopus *redundant genes remain to be determined.

## Methods

### Targeting of *Pygo1 *and *Pygo2*

*Pygo1 *and *Pygo2 *genes were each targeted by flanking the critical coding regions of the third exon containing the PHD motif with LoxP sequences. Targeting constructs were made using the Flp/Cre vector previously described [34], which carries a neomycin-resistance gene flanked by FRT sequences, and three unique restriction sites for subcloning the two blocks of homology for driving recombination, and the critical region to be flanked by LoxP sequences. For each construct, the three genomic segments required for subcloning were made by PCR from RI ES cell DNA. The resulting targeting constructs were confirmed by sequencing.

For *Pygo1*, the genomic sequences used for PCR were: 5' forward, GTGAAGGAGAGATGGATAAGTATG; 5' back, TAGACCCTAACCACCTACAAG; exon forward, GGTTAGGGTCTATGTGCTGG; exon back, TCACCAAATCTCTGTTCTACAC; 3' forward, TGTGTAGAACAGAGATTTGGTG; 3' back, CAGTGAAGAAAGAGGGTCAG. For *Pygo2*, the genomic sequences used for PCR were: GCCTGGGTTGCTTGTCTTCTG and CCACCTTACTTGTGTGTGAGGATACATAC, CCAAGTCCCAGCATCTCTTAC and CCAGTCATACCAGCAACAAG, and exon sequences TGGGTGCTGGGAACAGAAC and CAACAACAACAGAAGACAAGC.

Linearized constructs were electroporated into RI ES cells, and resulting targeted ES cells used for C57/Bl6 blastocyst injections according to standard protocols. Resulting chimeras were mated with Swiss Black mice, and the targeted stocks maintained on a mixed 129/Swiss Black background.

Germline null alleles of both *Pygo1 *and *Pygo2 *were generated by mating heterozygous floxed mice with the CMV-Cre mice [15]. The sequences of the primers used for genotyping PCR were: *Pygo2 *null allele, forward (F) CCTGGATTCTTGTTGCTGGTATG; reverse (R) AAGGTATTTGGTGCTCCGAGGG; *Pygo2 *WT or floxed allele, F TGTCTTGATGACAGCGTTTAGCC, R AGATTCAGTAAGCTGAGCCTGGTG; *Pygo1 *null allele, F AGTTTGAAATAGCGACGAGTTTGAG, R 5'-CACTTCTGCCCCTCTCTTTGC; *Pygo1 *WT or floxed allele, F AAGCGTGCCTCATCTCCATCCCTAAG, R GCCCTCCCCGACGTTTATATTG.

The noon of the day that vaginal plugs were observed was designated E0.5.

### Confocal microscopy

Kidneys were dissected, fixed in paraformaldehyde, treated with methanol, and washed with PBS containing Tween-20 (PBS-T) prior to treatment with lectins and antibodies. PBS-T was used for incubations of the tissues with fluoroscein-conjugated *Dilichos biflorus *aggutinin (DBA, Vector; Burlingame, USA), and PBS-T plus 2% goat serum was used for incubations with the antibodies. The primary antibodies were anti-WT1 (c-19; Santa Cruz Biotechnology, Santa Cruz, CA, USA), anti-uvomorulin (e-cadherin, Cdh1, Sigma-Aldrich St. Louis, MO, USA), and anti-Cited1 (Neomarkers-Lab Vision Corporation, Fremont, CA, USA). The secondary antibodies were Alexa 555-conjugated anti-rabbit and Alexa 633-conjugated anti-rat antibodies (Molecular Probes, Eugene, OR, USA).

The tissues were imaged with a laser scanning microscope (Carl Zeiss, Thornwood, New York, USA) equipped with an argon (488 nm) and two HeNe lasers (543 nm and 633 nm). Optical sections approximately 2 μm thick were obtained every 5 μm to a depth of at least 65 μm. The sections began at the surface of the kidney and were on a plane tangential to it. Two Z-stack series were obtained, one from each of the two kidneys of each embryo. Ureteric bud tips identified by section tracing were counted within a defined area of the confocal image.

### *In situ *hybridization

Whole-mount *in situ *hybridization was performed as previously described [35]. Riboprobes to *Wnt11 *and *Wnt7b *were described previously [35]. The *Wnt9b *riboprobe was provided by T. Carroll [2].

### Pygo1 and Pygo2 antibody production

To generate anti-human Pygo2 (anti-hPygo2) and anti-mouse Pygo1 (anti-mPygo1) polyclonal antibodies, we subcloned cDNA by PCR corresponding to amino-acid residues 80–327 of human Pygo 2 or amino-acid residues 76–263 of mouse Pygo1 into pGEX4T1 (Amersham Health, Piscataway, NJ, USA). The PCR fragments of hPygo 2 and mPygo 1 lack both NHD and PHD conserved regions of hPygo2 and mPygo1. GST-hPygo2 and GST-mPygo1 proteins were expressed in bacteria, purified, and injected into rabbits for antibody production by (Proteintech Group Inc., Chicago, IL, USA). The rabbit antisera of anti-mPygo1 and anti-hPygo2 were initially allowed to bind to the GST affinity matrix to remove any antibodies against GST. The anti-hPygo2 and anti-mPygo1 antisera were then separated from the GST affinity matrix and allowed to bind to the GST-hPygo2 or GST-mPygo1 affinity columns, respectively. The bound antibodes were eluted with elution buffer. To further ensure antibody specficity, the purified antibodies were extensively incubated with *Pygo1*/*2 *double-homozygous mutant embryo extract before use.

### BAT-gal transgene reporter assay of canonical Wnt signaling

X-gal staining of both embryos and developing kidneys was performed as previously described [21]. Care was taken to reduce endogenous β-galactosidase activity within the developing kidney by increasing pH of the X-gal staining solution to 8.0. Changes in transgene β-Gal expression were quantitated using a β-Gal ELISA (Enzyme-linked immunoassay) kit (Roche, Indianapolis, IN), normalizing according to total protein. Each genotype was represented by a sample size of 4 except the non-transgenic (*n *= 5), *Pygo1*^+/- ^; *Pygo2*^+/+ ^(*n *= 6), *Pygo1*^-/- ^; *Pygo2*^+/- ^(*n *= 8), and *Pygo1*^-/- ^; *Pygo2*^-/- ^(*n *= 6) groups.

### Microarray analysis

Total RNA was isolated from E18.5 kidneys dissected from normal and *Pygo1*/*Pygo2 *compound null embryos using a commercial kit (Stratagene Absolutely RNA Microprep Kit; La Jolla, CA, USA). An aliquot (300 ng) of total RNA was processed and labeled using a commercial kit (TargetAmp 1-Round Aminoallyl-aRNA Ki; Epicentre, Madison, WI, USA). Labeled RNA was hybridized to microarrays (Sentrix Mouse-6 expression Beadchip; Illumina, San Diego, CA) providing coverage of over 47000 genes and expressed sequence tags as previously described [36]. Microarray analysis of Pygo1/Pygo2 null and normal wild-type kidneys was performed in biological triplicate. Raw signal intensities of each probe were obtained from data analysis software (Beadstudio ;Illumina) and imported into GeneSpring GX 7.3 (Agilent Technologies, Palo Alto, CA, USA). Genes were selected on the basis of greater than two-fold average fold change and statistical significance (*p*-value < 0.05). Previously described Wnt target genes were obtained from .

### Quantitative PCR validation of microarray results

Total RNA from E18.5 Pygo1/Pygo2 null and control kidneys (both represented in duplicate and distinct from the kidneys used for microrray analysis) was purified using a commercial kit (Absolutely RNA Microprep Kit; Stratagene, La Jolla, CA USA) including DNAse1 treatment. cDNA was generated using random hexamers according to conventional protocols (Invitrogen, Carlsbad, CA, USA). The following primers were generated to include the sequence obtained from the Illumina probe: Actb (TTGCTGACAGGATGCAGAAG, ACATCTGCTGGAAGGTGGAC); Aldh1a7 (CCAGAAAGTGGTGTTTTGCT, GAGTTACAGAGAGCTTGCACCTT); Col8a1 (GCAGACAGGCATCTTCACCT, TGTGTACATCATGGGCTCGT); Csrp1 (CAGCATAGCCCAGGGTAGAG, TGGGCAAGGTAGTGAAGGTT); Klk5 (GCAGCATTACACCCGTCATA, TTGCCTCCATCCATATCTCC); Picalm (GGGAGGGAACAGAAATCCTT, GCACCGATCAACAGTGCAG); Pygo2 (TTCTGGGAACTTGTGCACTG, AACTTCCTCCAGCCCATTTT); Ren2 (TTGTGAGCTGTAGCCAGGTG, TGTGCACAGCTTGTCTCTCC) and Tia1 (TGATTGAAGGGCTACTAGAGTGGT, AGCCCCAGAGGACAATTTTT) using Primer3 software [37]. Relative quantitative PCR was performed according to the conventional SYBR Green protocol (Stratagene) using a quantitative PCR system (Mx3000p; Stratagene). Dissociation curve and agarose-gel analysis of each primer set were used to insure specificity of the amplicon. All data were normalized to an internal housekeeping control (Actb) and analyzed using the 2(-Delta Delta C(T)) method [38].

## Authors' contributions

SP designed and did most of the work for targeting the Pygo genes, provided oversight for the project and helped write the paper. KS did most of the experiments, including confocal analysis with LP, in situ hybridizations with HH, and helped write the paper. XL made and tested the Pygo antibodies. NS and RL provided useful suggestions and helped with the immunostaining and Bat-Gal analyses.
